# Angiomyolipoma of Uterine Cervix: Report of a Rare Case

**DOI:** 10.7759/cureus.38950

**Published:** 2023-05-12

**Authors:** Nfn Kiran, Raghunath Ramanarasimhaiah, Shahbaz Khan, Kokila Mody

**Affiliations:** 1 Pathology and Laboratory Medicine, Northwell Health, Staten Island, USA; 2 Gastrointestinal, Hepatobiliary and Transplant Pathology, Indiana University School of Medicine, Indianapolis, USA; 3 Hematopathology, Northwell Health, New York, USA; 4 Pathology, University of Oklahoma Health Sciences Center, Oklahoma City, USA

**Keywords:** smooth muscle tumor, desmin expression, smooth-muscle actin, angiomyolipoma’s, uterine lipoleiomyoma

## Abstract

Angiomyolipoma (AML) is classified as perivascular epithelioid cell neoplasm (PEComas) and is commonly seen in the kidney. AML is a solid mesenchymal neoplasm rarely encountered at the extrarenal site. Extrarenal AML is infrequently seen in the female genital tract. Four cases of AML of the cervix have been reported in the literature to our knowledge. We report a case of a 44-year-old female patient who presented with complaints of “lower abdominal pressure” and a history of post-coital bleeding and human papillomavirus (HPV) infection. A cyst in the uterine cervix was found incidentally on computerized tomography (CT) scan of the abdomen and pelvis. The patient underwent a loop electrosurgical excision procedure. The histologic and immunohistochemical features of the cervical biopsy favored the diagnosis of AML. The patient underwent a laparoscopic hysterectomy with bilateral salpingectomy. Grossly, a 4 cm white soft-to-firm mass was identified within the anterior lip of the cervix. Microscopy of the mass showed smooth muscle proliferation with prominent blood vessels, and scant mature adipose tissue trapped in between the smooth muscle bundles. Immunohistochemical stains showed smooth muscle actin (SMA) and desmin highlighting the smooth muscle component of AML. The histology and immunohistochemistry of the cervical mass in the surgical specimen were identical to the biopsy specimen and a diagnosis of AML was made.

## Introduction

Angiomyolipoma (AML) is a benign, solid, mesenchymal tumor and is one of the perivascular epithelioid cell neoplasms (PEComas) [1-2]. It is the most common benign renal tumor but rarely encountered at extrarenal sites, the liver being the second commonest site [3-4], and is usually an incidental imaging finding [5]. AMLs most commonly involve the uterus within the female genital tract [6].

Usually, AML occurs sporadically but an association with tuberous sclerosis has been reported [7]. Angiomyolipoma is typically composed of variable percent of three elements: blood vessels (vascular endothelial cells), smooth muscle (cells), and mature adipose tissue (mature adipocytes) [8-9]. This benign neoplasm, often found in the kidney, has been described as the "most controversial of benign tumor and tumor-like lesions in the kidney" by Price and Mostofi [10]. The malignant variant of angiomyolipoma is known as epithelioid angiomyolipoma (EAML), rarely encountered within the kidney [11]. Mutations in TSC1 and TSC2 genes increase the risk of developing renal AML [12-13]. Large tumors can present with symptoms of abnormal vaginal bleeding, pelvic pain, and abdominal bloating, and most small-sized tumors are asymptomatic [14]. 

## Case presentation

**Figure 1:**
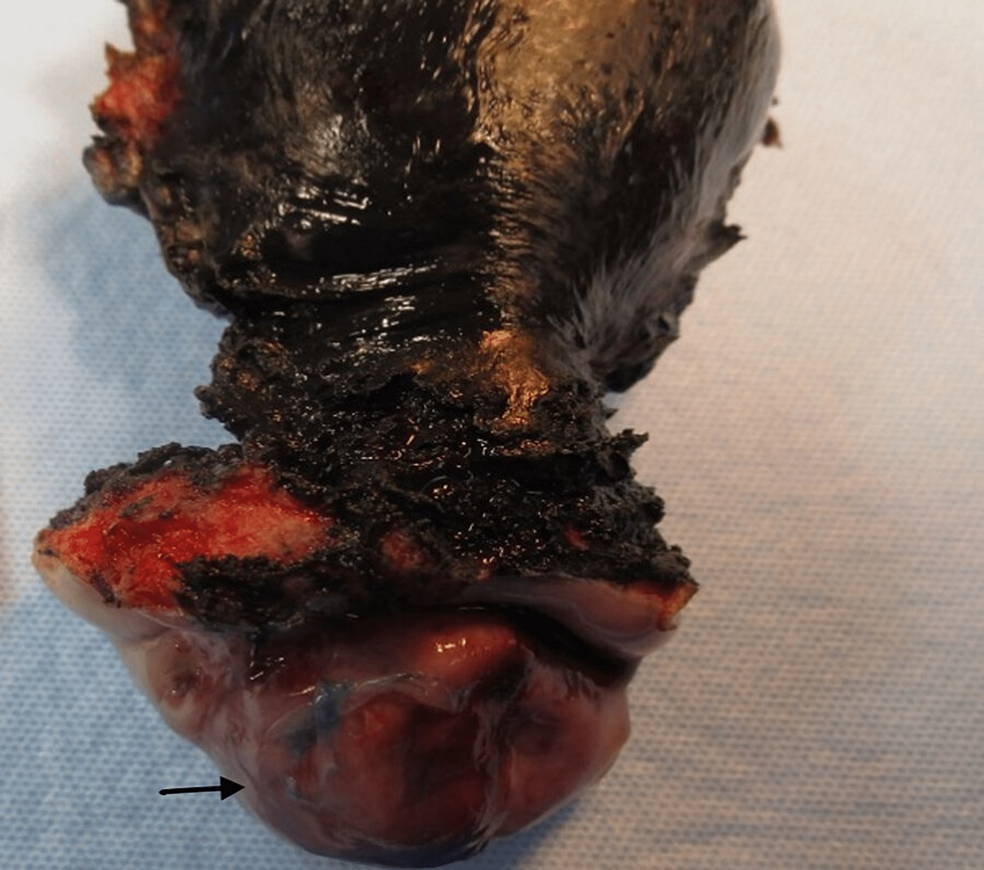
Gross image of the uterine cervix revealed a mass present within the anterior lip of the cervix (black arrow)

A 44-year-old female patient presented with complaints of “lower abdominal pressure” and a history of post-coital bleeding and Human papillomavirus (HPV) infection. A computerized tomography (CT) scan of the abdomen and pelvis revealed an incidental finding of a cyst in the uterine cervix. The patient underwent a loop electrosurgical excision procedure (LEEP), and a biopsy from the “cervical lesion at 12 o clock” was collected. The histologic and immunohistochemical features of the biopsy specimen helped to make a diagnosis of AML. The patient underwent a laparoscopic hysterectomy with bilateral salpingectomy. Gross examination revealed a 4 x 3 x 2 cm white soft-to-firm mass present within the anterior lip of the cervix (Figure 1). 

**Figure 2:**
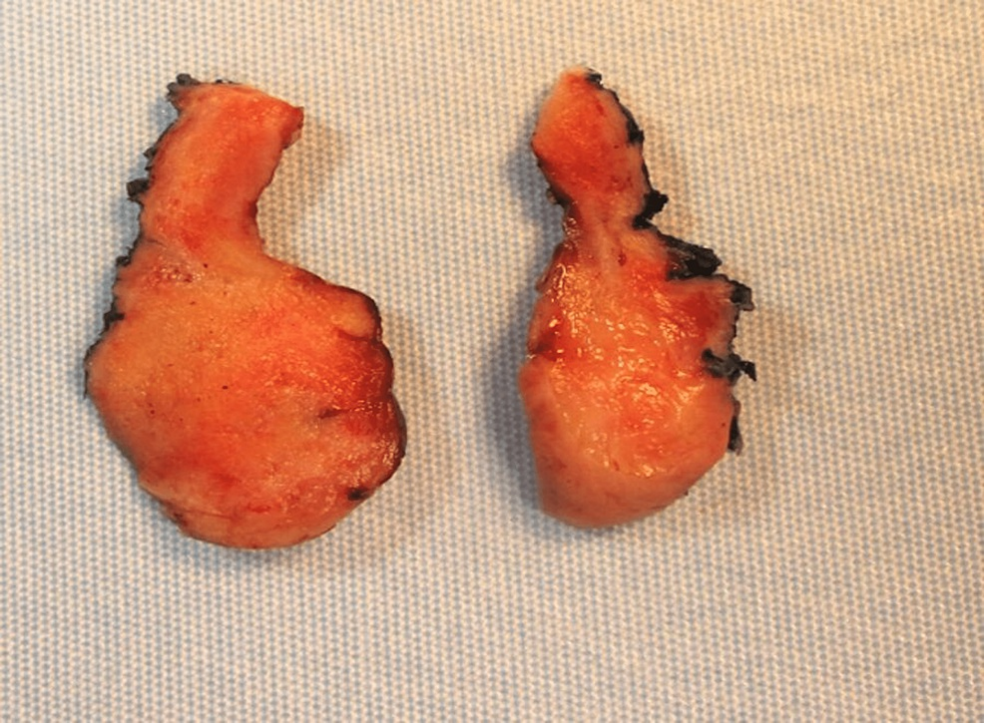
Gross image, cross-section of the cervical mass with a tan-white smooth cut surface

Sectioning reveals a tan-white smooth cut surface (Figure 2).

**Figure 3:**
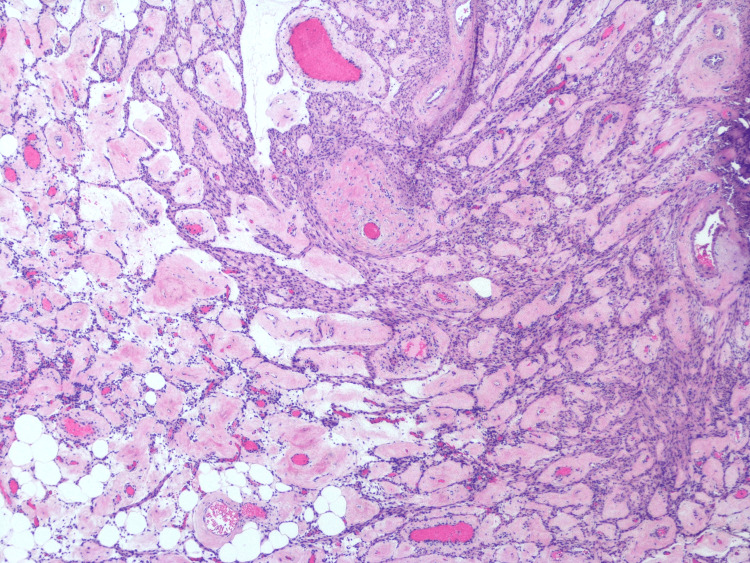
Microscopy of cervical mass (hematoxylin and eosin stain, 40x)

**Figure 4:**
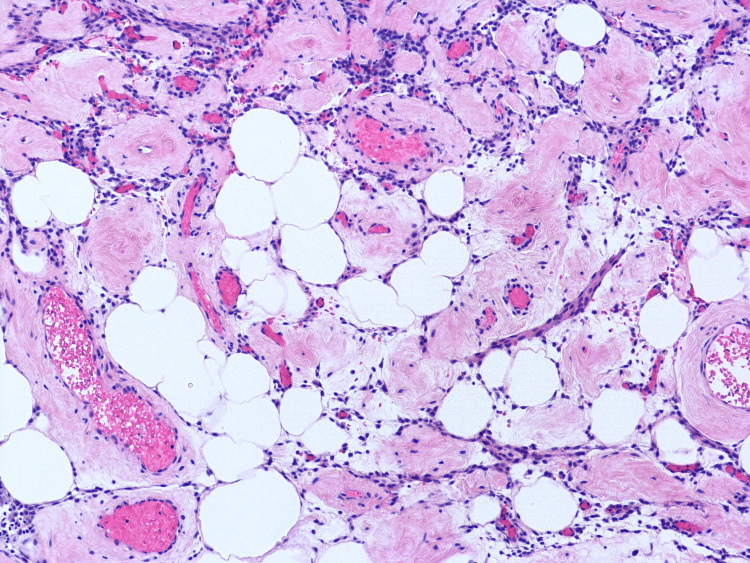
Microscopy of cervical mass (hematoxylin and eosin stain, 100x)

Microscopy of the cervical mass revealed smooth muscle proliferation with prominent blood vessels, patchy hyalinization, and scant mature adipose tissue trapped between smooth muscle bundles (Figures 3, 4).

**Figure 5:**
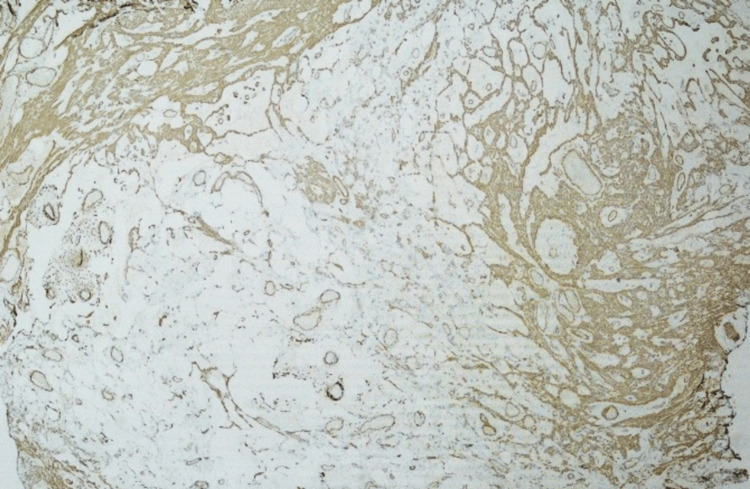
Immunohistochemistry - smooth muscle actin stain, 20x

Immunohistochemical stains showed smooth muscle actin highlighting smooth muscle components (Figure 5).

**Figure 6:**
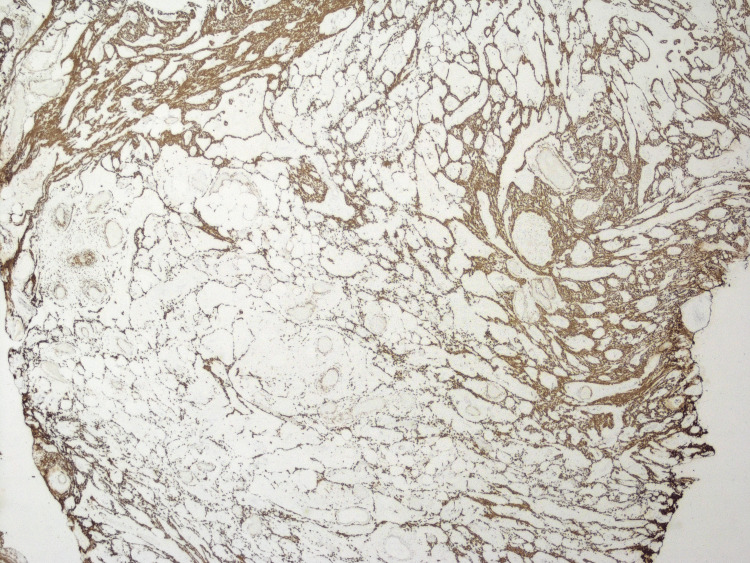
Immunohistochemistry - desmin stain, 20x

 Desmin highlighted the smooth muscle component (Figure 6).

The specimen was negative for human melanoma black (HMB-45). The histomorphology and immunohistochemical profile of the lesion in the surgical specimen was identical to the biopsy specimen. The diagnosis of AML of the uterine cervix was confirmed.

## Discussion

Angiomyolipoma (AML) is a benign tumor that predominantly arises in the kidney and rarely as an extrarenal tumor. The distinctive histological features of AML, characterized by mature adipose tissue, tortuous thick-walled blood vessels, and smooth muscle cells arranged in sheets or other patterns, were described several decades before the term "angiomyolipoma" was coined by Morgan et al. in 1951 [15].

AML is composed of haphazardly arranged mature adipose tissue, smooth muscle cells, and tortuous, thickened blood vessels. AML can occur in two forms: sporadic (most common, with a female predominance) and hereditary (with no gender predilection). AML is commonly associated with tuberous sclerosis (TSC), and renal AML is the most common manifestation of renal disease in patients with TSC. Mutations in TSC1 and TSC2 genes increase the risk of developing renal AML [12-13]. Association with sporadic lymphangioleiomyomatosis (LAM) in 60% was seen [16]. Renal and other extra-renal AML smooth muscle cell shows immunoreactivity to HMB-45 as compared to non-vascular smooth muscle cells [17]. Non-vascular smooth muscle cells of AML are positive for α-smooth muscle actin, desmin, vimentin, and progesterone receptor (PR) and negative for cytokeratin, CD34, S-100, and estrogen receptor (ER) [18]. 20% of angiomyolipomas are negative for HMB-45 [19]. HMB-45 antigen staining is helpful in the diagnosis of uterine smooth muscle tumors. 

Differential diagnoses include lipoleiomyoma and vascular leiomyoma with the fat component. Lipoleiomyomas commonly occur in postmenopausal women aged 50-60 years and are rarely found in the cervix [20]. The pathogenesis includes perivascular immature proliferating mesenchymal cells giving rise to fat cell components or adipocytes formed from smooth muscle cell transformation by intracellular deposits of lipids [19]. AML has thick-walled blood vessels on microscopy as compared to lipoleiomyoma; it has less significant vascular prominence. Vascular leiomyomas with mature adipocytes are rare and may mimic angiomyolipoma. Vascular leiomyoma is a subtype of leiomyoma and contains thick-walled vessels. The pathological feature includes vascular spaces lined by endothelial cells [21]. The treatment of symptomatic AML of the uterine cervix includes myolysis, radiofrequency ablation, radical hysterectomy, and tumor embolization [14]. 

While the kidney is the most common location for AML, extrarenal AMLs are extremely rare. Only four cases of AML of the uterine cervix have been reported [22].

## Conclusions

Angiomyolipoma occurring in the uterine cervix is very rare. To date, only four cases have been reported. Angiomyolipoma should be considered as one of the differential diagnoses in the case of lower abdominal mass and dysfunctional uterine bleeding. A careful and detailed histologic investigation is necessary to alleviate the concerns of both the patient and the surgeon. Reporting this extremely rare and interesting case will definitely help both pathologists and clinicians to diagnose and treat this benign condition.

## References

[1] Djerroudi L, Masliah-Planchon J, Brisse HJ (2023). Metastatic malignant perivascular epithelioid cell tumors with microsatellite instability within Lynch syndrome successfully treated with anti-PD1 pembrolizumab. JCO Precis Oncol.

[2] Okamoto K, Okada Y, Ohno K (2018). A rare case of perivascular epithelioid cell tumor (PEComa) of the greater omentum. World J Surg Oncol.

[3] Zhou H, Guo M, Gong Y (2017). Challenge of FNA diagnosis of angiomyolipoma: a study of 33 cases. Cancer Cytopathol.

[4] Baumgartner E, Garapati M, Sanders R, Eloubeidi M, Rosenblum F (2022). Fine needle aspiration of hepatic angiomyolipoma with extramedullary hematopoiesis: a case report. Cytopathology.

[5] Vos N, Oyen R (2018). Renal angiomyolipoma: the good, the bad, and the ugly. J Belg Soc Radiol.

[6] Huang PC, Chen JT, HO WL (2000). Clinicopathologic analysis of renal and extrarenal angiomyolipomas: report of 44 cases. Zhonghua Yi Xue Za Zhi (Taipei).

[7] Bouaziz H, Ghalleb M, Tounsi N (2021). A renal angiomyolipoma with a challenging presentation: a case report. J Med Case Rep.

[8] Wang F, Wang H (2020). Case report: rare presentation of primary angiomyolipoma in the middle ear. BMC Surg.

[9] Wroclawski ML, Baccaglini W, Pazeto CL (2018). Extrarenal angiomyolipoma: differential diagnosis of retroperitoneal masses. Int Braz J Urol.

[10] Fejes Z, Sánta F, Jenei A, Király IE, Varga L, Kuthi L (2022). Angiomyolipoma of the kidney-clinicopathological analysis of 52 cases. Pathol Oncol Res.

[11] Guo B, Song H, Yue J, Li G (2016). Malignant renal epithelioid angiomyolipoma: a case report and review of the literature. Oncol Lett.

[12] Zhang N, Wang X, Tang Z (2020). The correlation between tuberous sclerosis complex genotype and renal angiomyolipoma phenotype. Front Genet.

[13] Venyo AK (2016). A review of the literature on extrarenal retroperitoneal angiomyolipoma. Int J Surg Oncol.

[14] (2023). Angiomyolipoma of Uterine Cervix. https://www.dovemed.com/diseases-conditions/angiomyolipoma-uterine-cervix/.

[15] Morgan GS, Straumfjord JV, Hall EJ Angiomyolipoma of the kidney. J Urol.

[16] Lienert AR, Nicol D (2012). Renal angiomyolipoma. BJU Int.

[17] Monteiro R, Sharma S, Gupta S (2019). Extrarenal angiomyolipoma in uterine cervix: rare presentation in unusual site. International Journal of Research in Medical Sciences.

[18] Yaegashi H, Moriya T, Soeda S, Yonemoto Y, Nagura H, Sasano H (2001). Uterine angiomyolipoma: case report and review of the literature. Pathol Int.

[19] Aung T, Goto M, Nomoto M, Kitajima S, Douchi T, Yoshinaga M, Yonezawa S (2004). Uterine lipoleiomyoma: a histopathological review of 17 cases. Pathol Int.

[20] Adaikkalam J (2016). Lipoleiomyoma of cervix. J Clin Diagn Res.

[21] An J, Cho HY, Kim NR (2010). Uterine vascular leiomyoma with a fat component mimicking angiomyolipoma: a case report. J Womens Med.

[22] Yilmaz M, Ingec M, Isaoğlu U (2013). Angiomyolipoma of the uterus. Eur J Gen Med.

